# *In Vivo* Activity and Pharmacokinetics of Nemorosone on Pancreatic Cancer Xenografts

**DOI:** 10.1371/journal.pone.0074555

**Published:** 2013-09-05

**Authors:** Robert J. Wolf, Ralf A. Hilger, Jörg D. Hoheisel, Jens Werner, Frank Holtrup

**Affiliations:** 1 Division of Functional Genome Analysis, German Cancer Research Center (DKFZ), Heidelberg, Germany; 2 Department of Internal Medicine (Cancer Research), University Hospital of Essen, Essen, Germany; 3 Department of General Surgery, University of Heidelberg, Heidelberg, Germany; Wayne State University School of Medicine, United States of America

## Abstract

Pancreatic cancer is one of the leading cancer-related causes of death in the western world with an urgent need for new treatment strategies. Recently, hyperforin and nemorosone have been described as promising anti-cancer lead compounds. While hyperforin has been thoroughly investigated *in vitro* and *in vivo*, *in vivo* data for nemorosone are still missing. Thus, we investigated the growth-inhibitory potential of nemorosone on pancreatic cancer xenografts in NMRI nu/nu mice and determined basic pharmacokinetic parameters. Xenograft tumors were treated with nemorosone and gemcitabine, the current standard of care. Tumor sections were subjected to H&E as well as caspase 3 and Ki-67 staining. Nemorosone plasma kinetics were determined by HPLC and mass spectrometry. Induction of CYP3A4 and other metabolizing enzymes by nemorosone and hyperforin was tested on primary hepatocytes using qRT-PCR. At a dose of 50 mg/kg nemorosone per day, a significant growth-inhibitory effect was observed in pancreatic cancer xenografts. The compound was well tolerated and rapidly absorbed into the bloodstream with a half-life of approximately 30 min. Different metabolites were detected, possibly resembling CYP3A4-independent oxidation products. It is concluded that nemorosone is a potential anti-cancer lead compound with good bioavailability, little side-effects and promising growth-inhibitory effects, thus representing a valuable compound for a combination therapy approach.

## Introduction

Despite many advances in the field of cancer research and treatment, pancreatic cancer remains one of the most challenging tumors, with its mortality rate almost matching the incidence rate [1]. This is mainly due to difficulties in diagnosing pancreatic cancer in an early resectable stage and its pronounced chemo-resistance. Thus, more than 80% of patients present with an advanced-stage tumor already metastasized to regional lymph nodes and the liver [2]. Gemcitabine, the current standard of care for pancreatic cancer, only moderately improves median survival times between 5 and 6 months [3], thus emphasizing the need to explore novel therapeutic compounds.

**Figure 1:**
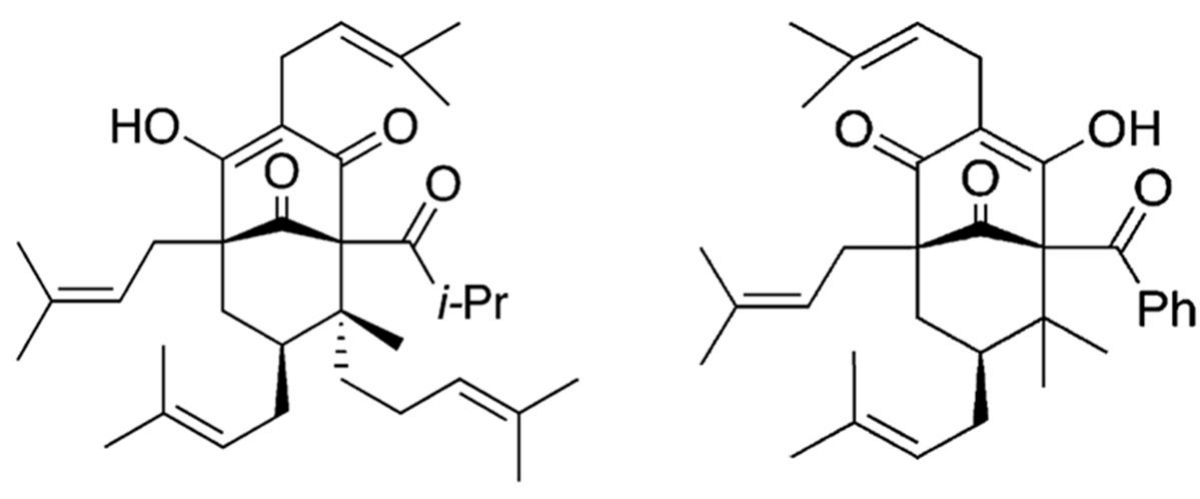
Hyperforin and nemorosone. Closely related chemical structures of hyperforin (left) and nemorosone (right), two polycyclic polyprenylated acylphloroglucinols.

Polycyclic polyprenylated acylphloroglucinols (PPAPs) are a class of compounds with various biological activities ranging from anti-depressant, anti-cancer and anti-inflammatory to anti-microbial activity [4]. Among them, hyperforin (Figure 1), a remedy from St. John’s wort ( *Hypericum* *perforatum* ), has gained public interest as a popular anti-depressant of natural origin with a novel mechanism of action [5]. It has also been shown to possess anti-cancer properties *in vitro* and *in vivo* [6–8]. However, via binding to the pregnane X receptor (PXR), hyperforin strongly upregulates the expression of CYP3A4, a member of the cytochrome P450 family involved in xenobiotics metabolism [9]. Thus, if combined with chemo-therapeutic drugs metabolized by CYP3A4, hyperforin would greatly reduce the efficacy of a potential anti-cancer combination therapy approach since it accelerates metabolization of the drug(s) it is combined with.

Being structurally related to hyperforin, pronounced anti-cancer properties have also been demonstrated *in vitro* for nemorosone (Figure 1) on cell lines of various origins, among them neuroblastoma and leukemia [10,11]. More detailed analyses of its mechanism of action on pancreatic cancer cells showed rapid elevation of cyctosolic calcium levels and depolarization of the mitochondrial membrane followed by activation of apoptosis via a stress response pathway known as the unfolded protein response [12]. Interestingly, differentiated normal cells were found to be 10-times less sensitive to a treatment with nemorosone, thus opening a potential therapeutic window to explore also its anti-cancer activity *in vivo*.

In this study, we demonstrate that, unlike hyperforin, nemorosone does not induce CYP3A4 and investigate its biological activity as well as basic pharmacokinetics *in vivo* in a pancreatic cancer xenograft model. It is shown that nemorosone is rapidly absorbed into the bloodstream and able to inhibit the growth of xenograft tumors.

## Materials and Methods

### Ethics Statement

This study was carried out in strict accordance with the recommendations in the Guide for the Care and Use of Laboratory Animals of the National Institutes of Health. The protocol was approved by the Committee on the Ethics of Animal Experiments of the regional council Karlsruhe, Germany (Permit Number: G-204/09). All surgery was performed under xylazine/ketamine anesthesia, and all efforts were made to minimize suffering.

### Implantation and treatment of xenograft tumors

Six to eight week old female athymic NMRI nu/nu nude mice (Charles River Laboratories, Sulzfeld, Germany) were housed in groups of 4 to 6 in individually ventilated cages (IVCs; Techniplast, Germany) at 22°C with a 12 h light/dark cycle and allowed to adapt to the housing conditions for one week before implantation of tumors. MIA-PaCa-2 cells were maintained as previously described [12], and 200 µl of a cell suspension (~2×10^6^) was injected s.c. into the right flank of each mouse to establish subcutaneous xenograft tumors. Tumor volume was regularly monitored using a digital caliper, and animals were randomly organized into treatment and control groups once mean tumor volume reached approximately 100 mm^3^.

Nemorosone, purified from methanolic flower extracts as reported earlier [12], was dissolved in dimethyl sulfoxide (DMSO) at 50 mg/ml, and gemcitabine hydrochloride (Eli Lilly, Germany) was dissolved in 0.9% NaCl solution. Injection solutions were sterile filtered and administered intraperitoneally (i.p.) using 0.3 ml insulin syringes (Becton Dickinson, Germany) after sterilization of the injection site. Body weight was controlled on a daily basis prior to i.p. injection. 1 µl/g body weight was injected to achieve a final concentration of 50 and 120 mg/kg for nemorosone and gemcitabine, respectively. Nemorosone was injected once daily whereas for gemcitabine the standard treatment schedule of every third day for four cycles was used [13].

### Immunohistochemical staining of xenograft tumors

One day after the last treatment, mice were sacrificed by cervical dislocation and xenograft tumors were resected, snap-frozen in liquid nitrogen and stored at -80°C. Tumors were mounted in a cryomicrotome (Leica, Germany) at -25°C and 5 µm slices were generated from tumor margins and centers to be transferred to glass slides.

For hematoxylin and eosine (H&E) staining, tumor sections were fixed for 5 s in acetone at -20°C, dried and stained in hematoxylin (Carl Roth, Germany) for 15 s and acidic 1.5% eosine in 96% EtOH (Merck, Germany) for 4 s. After dehydration by subsequent incubation in 70%, 96% and 2 × 100% EtOH, staining was fixed in RotiClear solution (Carl Roth, Germany) and cover glasses were mounted in paramount mounting medium (DAKO, USA).

For immunohistochemical staining with Ki-67 and active caspase 3 antibodies, sections were blocked with 3% peroxidase in methanol after fixation in cold acetone and washing in tris-buffered sline (TBS) supplemented with 0.1% (w/v) bovine serum albumine (BSA). Following another washing step, sections were incubated overnight at 4°C in 1:100 dilutions of rabbit anti-human Ki-67 or active caspase 3 antibodies (DAKO, USA) in TBS/0.1% BSA. Slides were washed in TBS/0.1% BSA supplemented with 0.1% (v/v) Tween-20 and subsequently in TBS/0.1% BSA before addition of horse-raddish peroxidase (HRP)-conjugated goat anti-rabbit IgG secondary antibody (DAKO, USA) for 45 min at room temperature. After washing, sections were stained with liquid 3,3' diaminobenzidine (DAB) chromogen diluted 1:500 in substrate buffer (DAKO, USA). Color development was monitored microscopically and stopped in dH_2_O. Sections were then counterstained in hematoxylin, dehydrated and mounted as mentioned above.

### Analysis of nemorosone pharmacokinetics

50 mg/kg nemorosone were administered i.p. and mice were anesthetized with 7 mg/kg xylazine and 100 mg/kg ketamine 5, 20, 40, 60, 80, 100 and 120 min after application of nemorosone. Blood was drawn into heparinized syringes by heart punctuation and subsequently centrifuged for 10 min at 16,000 × g to prepare plasma. Nemorosone was extracted from plasma by adding 400 µl acetonitrile (ACN) to 100 µl plasma and centrifugation at 21,000 × g for 5 min at room temperature. The supernatant was transferred into a new vial and concentrated in a vacuum concentrator. The resulting pellet was dissolved in solvent for high pressure liquid chromatography (HPLC) analysis (50% H_2_O, 30% MeOH, 20% ACN). 10 µl of each sample were injected for analysis on a Waters 2690 HPLC System (Waters, Germany) equipped with a 996 PDA and a ZQ2000 ESI-MS detector (Waters, Germany). Separation was done using a C18 symmetry reversed-phase column (5 µm pore size, 4.2 × 250 mm; Waters, Germany) and a constant flow rate of 1 ml/min using a solvent gradient. Nemorosone absorbance was detected at 303 nm.

Nemorosone peak area in the chromatogram was quantified with the help of a calibration curve (Figure S3) derived from human plasma samples spiked with defined concentrations of nemorosone (10 ng/ml to 100 µg/ml). HPLC chromatograms were analyzed using the Empower 2 software, and nemorosone plasma concentration kinetics after i.p. injection were fitted using TopFit 2.0 [14] under the assumption of a two-compartment disposition following a one-segment absorption.

### Analysis of expression changes of metabolizing enzymes in primary human hepatocytes

About 7.5 ×10^5^ primary human hepatocytes (Zen-Bio, NC, USA) were plated in each well of a collagen I-coated 12-well cell culture plate in plating medium (Zen-Bio, NC, USA) and allowed to attach for 10 h. Plating medium was exchanged with maintenance medium (Zen-Bio, NC, USA) and cells were grown to 80% confluence prior to incubation with 0.5 and 1 µM nemorosone and hyperforin dicyclohexylammonium salt (Sigma Aldrich, Germany) or 10 µM rifampicin (Sigma Aldrich, Germany) for 48 h. Cells were then harvested by trypsinization and total RNA was isolated with an RNeasy Mini kit (Qiagen, Germany) according to manufacturer’s instructions. RNA was subjected to quantitative real-time PCR analysis using the QuantiFast SYBR Green RT-PCR kit (Qiagen, Germany) and QuantiTect primers (Qiagen, Germany) as described previously [12].

### Statistical tests

All experiments were done at least in triplicates unless stated otherwise. For all experiments, Student’s t-test (in SigmaPlot 10.0) was used for significance analysis of the difference between two groups.

## Results

### Nemorosone does not induce CYP3A4 expression in primary human hepatocytes

Metabolization is an important part of the pharmacokinetic properties of a drug. Most xenobiotics are metabolized by the cytochrome P450 (CYP) family in the liver after being detected by nuclear receptors [15–17]. Being structurally very similar to nemorosone, hyperforin (Figure 1) has been demonstrated to induce CYP3A4 expression by binding the pregnane X nuclear receptor (PXR) and, thus, a faster metabolism of drugs co-administered with hyperforin [9,18]. In order to test whether also nemorosone is detected by the PXR and induces the expression of CYP3A4 as well as other important drug-metabolizing enzymes, primary human hepatocytes were cultured in the presence of nemorosone, hyperforin and rifampicin, a drug known to induce CYP3A4 expression by binding to the PXR receptor (positive control). After 48 hours of incubation, RNA was extracted from hepatocytes and subjected to qRT-PCR analysis to assess the expression of selected enzymes. 

**Figure 2:**
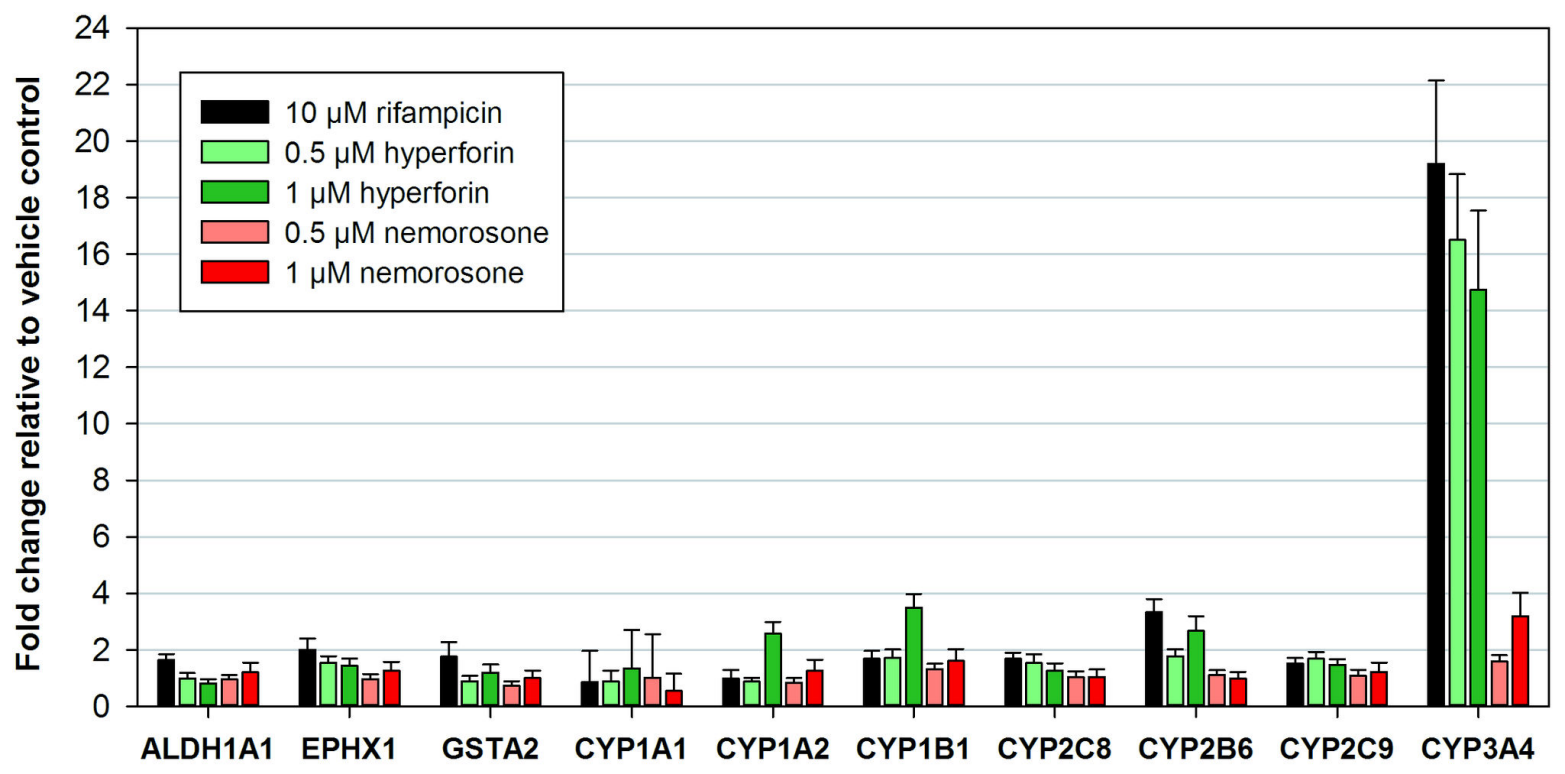
Induction of selected genes involved in metabolism. Primary human hepatocytes were treated with the indicated concentrations of rifampicin, hyperforin, nemorosone or vehicle only. RNA was extracted after 48 h and subjected to qRT-PCR analysis to detect expression changes of selected genes involved in drug metabolism. Expression values are relative to the vehicle control and represent means ± SD of triplicate measurements.

Figure 2 shows that treatment of human hepatocytes with hyperforin and rifampicin led to a significant up-regulation of *CYP3A4* gene expression of 15 to 20-fold as compared to the vehicle control. In contrast to that, treatment with 1 µM nemorosone only induced *CYP3A4* expression 3-fold, whereas there was almost no induction upon treatment with 0.5 µM nemorosone. Hyperforin treatment also slightly up-regulated *CYP1A2*, *CYP1B1* and *CYP2B6* expression, but there was no change detectable for the other tested metabolizing enzymes like aldehyde dehydrogenase 1A1 (ALDH1A1), epoxide hydrolase 1 (EPHX1) or glutathione S-transferase A2 (GSTA2). Thus, it can be hypothesized from these data, that hyperforin is mainly metabolized by CYP3A4 after being sensed by the PXR. Although being structurally similar to hyperforin, nemorosone does not seem to bind the PXR and, thus, metabolizing enzymes are only weakly induced rendering nemorosone potentially useful in an anti-cancer combination therapy approach.

### Nemorosone inhibits growth of xenograft tumors in vivo

**Figure 3:**
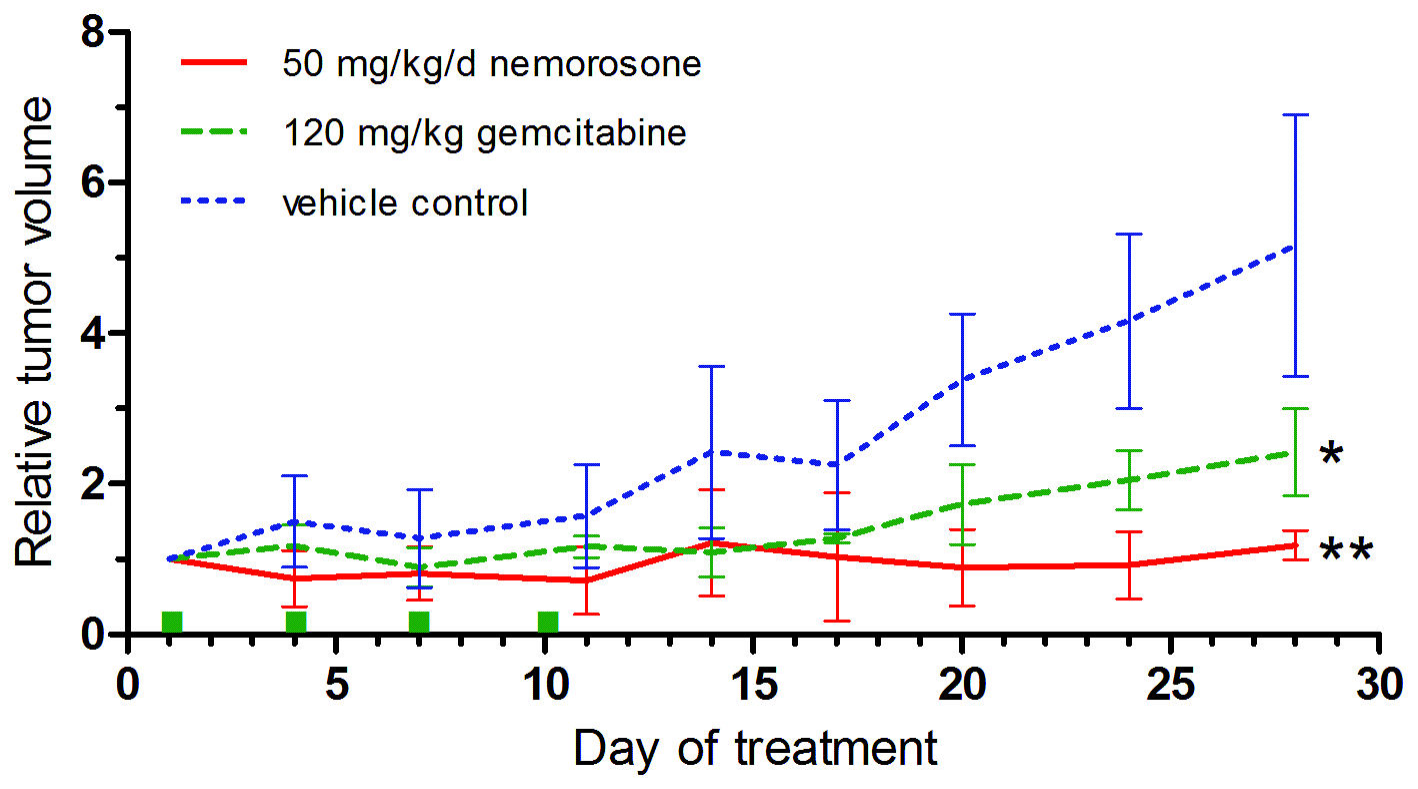
Tumor volume and body weight development in MIA-PaCa-2 xenograft mice. Mice were treated with daily i.p. injections of 50 mg/kg nemorosone, vehicle only or 120 mg/kg gemcitabine at the indicated time points (green dots). Tumor volume was measured 2-3 times per week using a digital caliper. Values represent the mean ± SD of 8 animals per group. * p < 0.05, ** p < 0.01 (compared to the vehicle control).

Figure 3 shows that daily injection of 50 mg/kg nemorosone resulted in a significant and complete abrogation of tumor growth as compared to the average of the control tumors which increased 5-fold in volume. A similar effect was seen in the control group receiving 120 mg/kg gemcitabine. At the same time, treatment with nemorosone demonstrated a good tolerability, since body weight remained stable over the treatment period of 28 days (Figure S1). However, it needs to be noted that animals receiving nemorosone started to hyperventilate shortly after injection. Normal respiration was restored again 5-10 min after i.p. application.

To examine histological effects of nemorosone treatment, animals were sacrificed after the treatment period and tumors were excised. Treated and control tissue was then stained for apoptosis and proliferation markers. 

**Figure 4:**
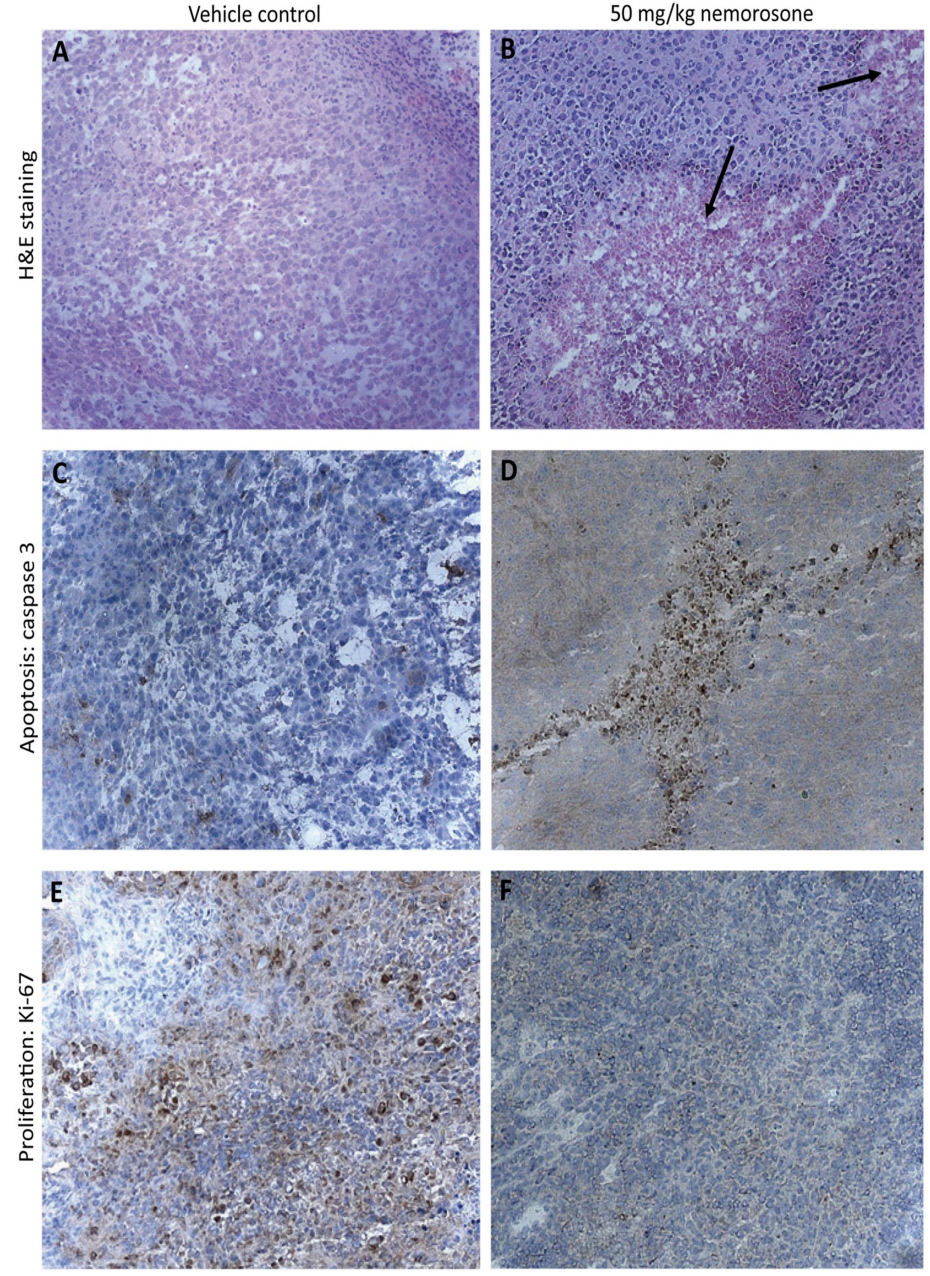
Hematoxylin-eosine and immunohistochemical staining of tumor sections. Sections of 5 µm of nemorosone-treated and control tumors were stained with hematoxylin-eosine (H&E; **A** and **B**) or immunohistochemically analyzed with antibodies directed against active caspase 3 (**C** and **D**) or Ki-67 (**E** and **F**). Treated tumors demonstrated reduced tumor mass due to apoptosis/necrosis (black arrows) and a lower number of proliferating cells as compared to the control (dark-brown cells).

Histological differences between treated and control tumors were visible in H&E-stained tumor sections (Figure 4). While control tumors exhibited a homogeneous distribution of viable cells, sections of nemorosone-treated tumors demonstrated large areas of necrotic or late-apoptotic cells characterized by eosine staining only. The absence of hematoxylin staining in these areas indicates karyolysis. Immunohistochemical staining using an antibody directed against active caspase 3 verified regions of excessive apoptosis (dark-brown cells) whereas control sections exhibited some background apoptosis only (Figure 4C and D). In areas lacking caspase-positive cells, the number of cells expressing the proliferation marker Ki-67 was found to be reduced in sections of nemorosone-treated tumors (Figure 4F). In contrast to this, a homogeneous distribution of proliferating cells characterized by a dark-brown staining was visible in control sections.

In summary, nemorosone treatment of MIA-PaCa-2 xenograft tumors *in vivo* was demonstrated to reduce the number of proliferating cells and induce apoptosis, thus leading to a reduction in tumor mass characterized by large regions of dying tumor cells.

### Pharmacokinetic analysis reveals rapid absorption and metabolization of nemorosone

Absorption and metabolization of nemorosone *in vivo* was assessed by withdrawing blood before and at different time points after i.p. injection of 50 mg/kg nemorosone. Nemorosone as well as potential metabolites were extracted from mouse plasma, and plasma concentration was analyzed for each time point by reversedphase high pressure liquid chromatography (RP-HPLC; detection at 303 nm).

**Figure 5:**
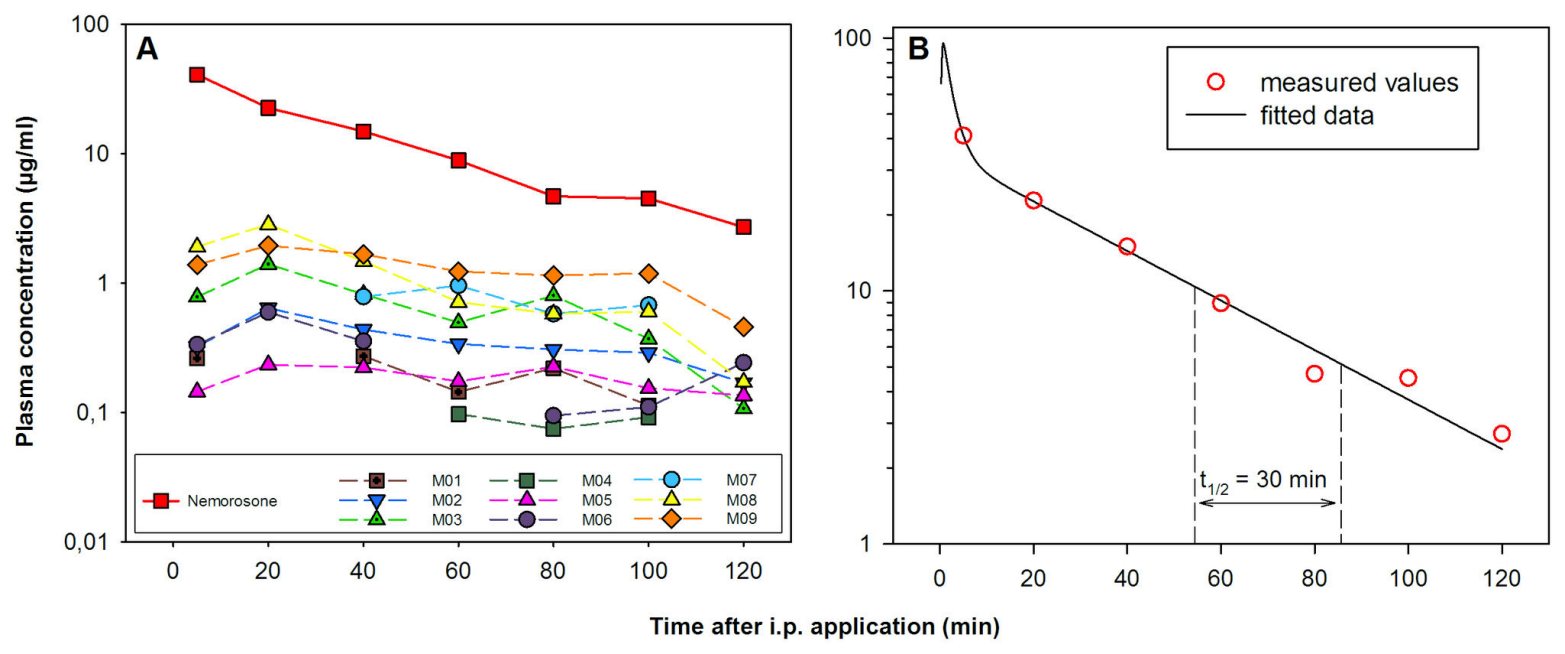
Plasma concentration of nemorosone and its metabolites. Plasma samples were taken at pre-defined time points after i.p. application of 50 mg/kg nemorosone. Nemorosone and its metabolites were quantified by RP-HPLC with the help of a calibration curve (Figure S3). (**A**) Plasma concentration of nemorosone (upper green line) and its potential metabolites (M01 to M09). (**B**) Pharmacokinetics of nemorosone were best described by a two-compartment model with a half-life of 30 min.

Five minutes post i.p. application, approximately 40 µg/ml (80 µM) nemorosone were detected in mouse plasma, pointing towards a very rapid absorption into the bloodstream (Figure 5A). At the same time point, 7 additional peaks resembling potential metabolites were found in the HPLC chromatogram (Figure 5A, Figure S2). However, plasma concentration of potential metabolites (M01-M09) was calculated to be 20 to 200-fold lower than that of nemorosone. Over the observed time of 120 min, nemorosone plasma concentration decreased logarithmically from 40 to 2.7 µg/ml with a half-life of approximately 30 min. Pharmacokinetics of nemorosone after i.p. application were found to be best described by a two-compartment disposition model yielding a biphasic line in the log-scale plot in Figure 5B. According to this model, nemorosone is rapidly absorbed and distributed in the bloodstream within the first 10 min after injection. 20 min after application, elimination seems to be the predominant process characterized by the straight fitted line. However, it needs to be noted, that the processes prior to the 5 min time point (absorption and distribution) were assumed and require further confirmation in a more detailed study. Thus, peak concentration within the first minutes could be much higher (~100 µg/ml).

Identity of nemorosone was further confirmed by an ESI mass spectrometry detector displaying molecule peaks for protonated nemorosone and its sodium and potassium salts (Figure S4). Furthermore, the mass of the molecule peaks of some metabolites was found to be increased by 16 u, suggesting that oxidation of nemorosone might be one of the main metabolization steps. However, due to the low resolution of the ESI-MS detector, the structure of nemorosone metabolites could not be characterized in detail.

## Discussion

Hyperforin and nemorosone are among the best studied members of the diverse class of polycyclic polyprenylated acylphloroglucinols and both are considered lead compounds for the development of anticancer therapeutics [4,6,12,19]. However, up to now anticancer activity *in vivo* has only been demonstrated for hyperforin but not for nemorosone [6]. Thus, this pilot study aimed at evaluating the activity of nemorosone against pancreatic cancer xenograft tumors *in vivo* as well as identifying basic pharmacokinetic parameters like absorption kinetics and metabolization.

Our results indicate that nemorosone potently inhibits growth of pancreatic cancer xenografts *in vivo* and seems to be even more effective than the current standard of care gemcitabine (Figure 3). While the administered nemorosone dose of 50 mg/kg per day was well tolerated over the treatment period, hyperventilation was observed directly after application of nemorosone indicating some systemic activity of this compound directly after absorption into the bloodstream. Similar observations were made for humulone, a structurally related compound isolated from hops, after intravenous injection into cats [20]. This indicates comparable pharmacodynamic properties which might be due to the action of these compounds on calcium homeostasis in muscles. This hypothesis is supported by the instantaneous nemorosone-induced disturbance of cellular calcium homeostasis observed *in vitro* [12].

Effects of nemorosone treatment on xenograft tumors were confirmed by H&E staining of tissue sections as well as immunohistochemically by staining active caspase 3 and the proliferation marker Ki-67. Nemorosone-treated MIA-PaCa-2 tumors exhibited more necrotic and/or apoptotic regions, which were associated with elevated caspase 3 activity and a lower number of proliferating cells expressing Ki-67, consistent with results obtained in MIA-PaCa-2 cells *in vitro* [12].

Measurement of plasma kinetics after i.p. injection of 50 mg/kg nemorosone confirmed bioavailability and absorption of the compound into the bloodstream (Figure 5). Five minutes after injection, nemorosone plasma concentration was found to be 40 µg/ml (80 µM) with logarithmic elimination dominating 20 min after injection and nemorosone demonstrating a plasma half-life of approximately 30 min. Measured values for plasma kinetics were best fitted assuming a two-compartment disposition model as also observed in a pharmacokinetic studies of hyperforin plasma concentration upon oral administration [21,22]. Calculated peak plasma concentration of nemorosone was ~100 µM. Thus, considering the high dose during the first 5-10 min after application, the observed acute hyperventilation can be explained by nemorosone reversibly interfering with healthy cells between peak plasma concentration and the onset of the elimination phase approximately 10 minutes later.

In total, 9 possible metabolites were detected in mouse plasma, most of them were already present at 5 min after i.p. injection (Figures 5 and S2). This points towards a rapid metabolization of nemorosone, consistent with the calculated half-life of only 30 min. Unfortunately, the structure of the metabolites could not be obtained due to the low resolution of the ESI-MS detector. However, considering that fact that the retention times observed in the RP-HPLC chromatogram are lower for all metabolites compared to nemorosone, oxidation or hydroxylation processes can be assumed. This assumption is further supported by the fact that, compared to nemorosone, the mass of selected metabolites is increased by 16 u, the molecular mass of oxygen (Figure S4). To obtain detailed structural information about the metabolites, an LC/ESI-MS system with higher resolution or a preparative approach for NMR analysis will need to be established. Both methods have been applied to hyperforin metabolized in rat liver microsomal systems and resulted in the identification of four hyperforin metabolites, each hydroxylated at the terminal methylene group of one of the four prenyl side chains [23]. The authors speculated that two rat cytochrome P450 isoforms ortholog to human CYP3A4 were mainly involved in the metabolization of hyperforin. This is consistent with the fact that hyperforin induces the expression of *CYP3A4 via* binding to the pregnane X receptor (PXR) [9,24]. *CYP3A4* expression was also found to be strongly induced by hyperforin and rifampicin, a substance known to activate PXR, upon treatment of human primary hepatocytes in this study (Figure 2). Interestingly, nemorosone was only moderately effective in inducing *CYP3A4* expression: Incubation of hepatocytes with 1 µM nemorosone only resulted in a 3-fold increase of *CYP3A4* mRNA compared to a 15-fold increase upon incubation with 1 µM hyperforin. This result is surprising considering the highly flexible ligand-binding cavity of the PXR which has been demonstrated to perfectly bind hyperforin [24,25]. Thus, given the close structural relationship to hyperforin, the PXR should be expected to also bind and induce *CYP3A4* expression at equimolar nemorosone concentrations. This does not seem to be the case, thus rendering higher concentrations of nemorosone for cancer treatment preferable over those of hyperforin for which multiple drug interactions related to CYP3A4 induction have been reported [9,18]. Considering the fact that an elevated PXR expression has also been observed in various pancreatic cells [26], treatment of pancreatic cancer with a combination therapy approach is more likely to be successful with nemorosone rather than hyperforin.

In conclusion, our study demonstrates a significant growth-inhibitory effect of nemorosone on pancreatic cancer xenografts while being well tolerated. Basic pharmacokinetic analysis suggests, that nemorosone is rapidly absorbed and metabolized via oxidation in a CYP3A4-independent manner. This makes nemorosone a very promising lead compound for combination therapies. Thus, more detailed studies on orthotopic xenograft models and pharmacokinetic analyses of higher resolution should be conducted.

## Supporting Information

Figure S1 **Body weight of treated and control mice.** Body weight of treated and control mice was routinely determined on a daily basis prior to i.p. injection. Values represent the mean ± SD of 8 animals per group.
(TIF)

Figure S2 **HPLC chromatograms of nemorosone and its metabolites detected in plasma.** Overlay of selected HPLC chromatograms (detection at 303 nm) of mouse (green) and human (blue) plasma samples as well as human plasma spiked with 100 ng/ml nemorosone (red) and mouse plasma 5 min after i.p. application of nemorosone (black) is shown.
(TIF)

Figure S3 **Standard row of human plasma spiked with increasing nemorosone concentrations.** A calibration line for nemorosone spiked into human plasma samples was calculated and run in parallel to the samples extracted from mouse plasma to allow quantification of nemorosone.
(TIF)

Figure S4 **ESI-MS spectra of nemorosone and selected metabolites.** ESI-MS spectra were recorded for each peak, and spectra for nemorosone as well as for metabolites M01, M06 and M08 are displayed. The molecule ion peaks of protonated nemorosone (m/z = 503.2) as well as its sodium (m/z = 525.2) and potassium (m/z = 541.1) salts are marked with black arrows. Potentially oxidized nemorosone sodium (m/z = 541.1) and potassium (m/z = 557.2) salts are marked with grey arrows.
(TIF)
