# Supplementary figures and images for: In Vivo Activity and Pharmacokinetics of Nemorosone on Pancreatic Cancer Xenografts
Source: PLoS One. 2013 Sep 5;8(9):e74555. doi: 10.1371/journal.pone.0074555 (PMC3764110; doi:10.1371/journal.pone.0074555)

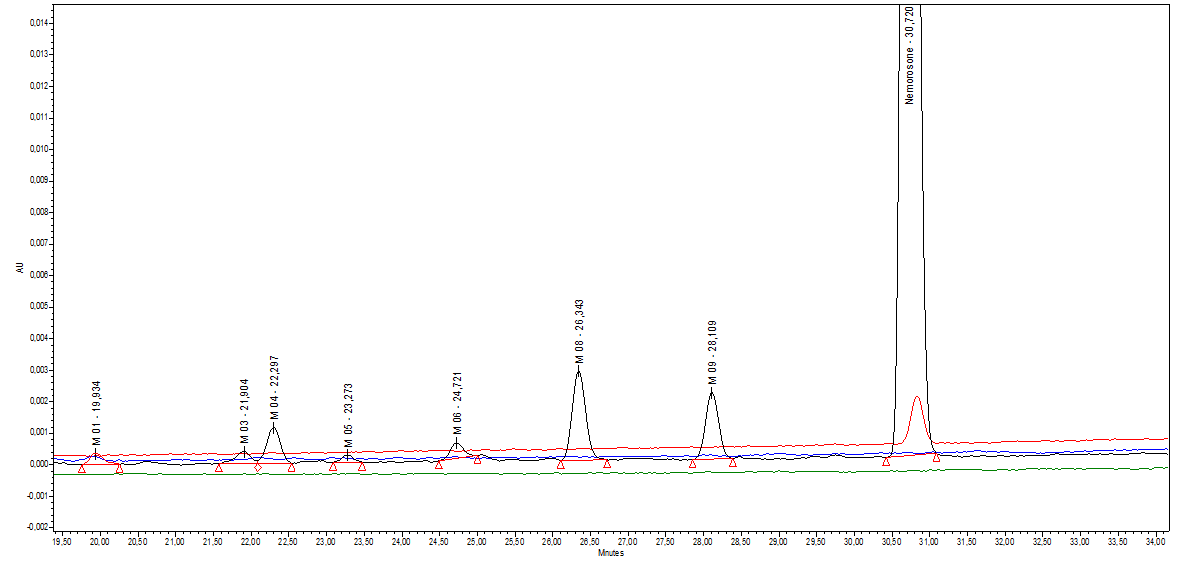

Supplement: Figure S2 — HPLC chromatograms of nemorosone and its metabolites detected in plasma. Overlay of selected HPLC chromatograms (detection at 303 nm) of mouse (green) and human (blue) plasma samples as well as human plasma spiked with 100 ng/ml nemorosone (red) and mouse plasma 5 min after i.p. application of nemorosone (black) is shown. (TIF) [file pone.0074555.s002.tif]

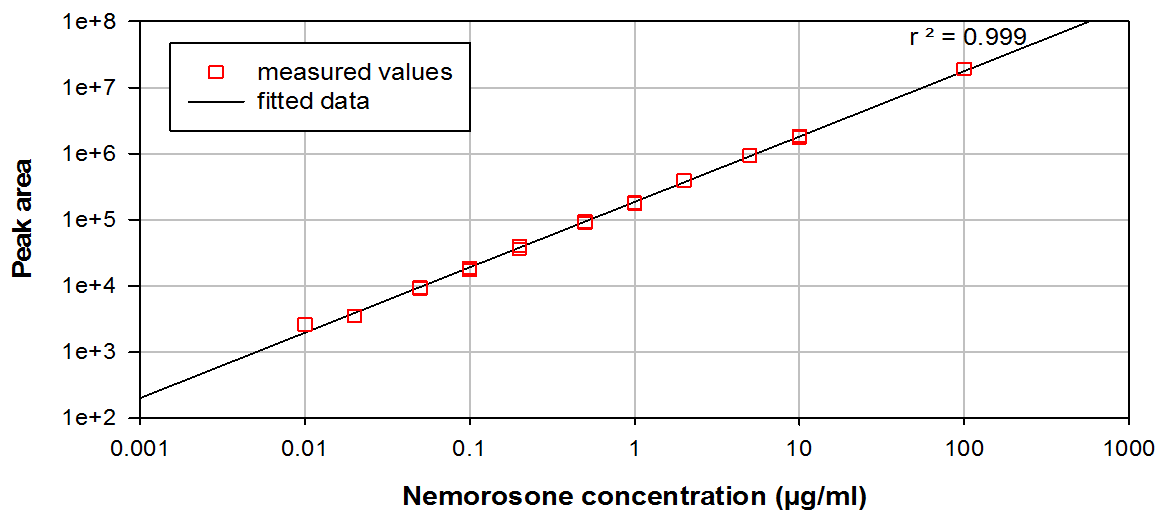

Supplement: Figure S3 — Standard row of human plasma spiked with increasing nemorosone concentrations. A calibration line for nemorosone spiked into human plasma samples was calculated and run in parallel to the samples extracted from mouse plasma to allow quantification of nemorosone. (TIF) [file pone.0074555.s003.tif]

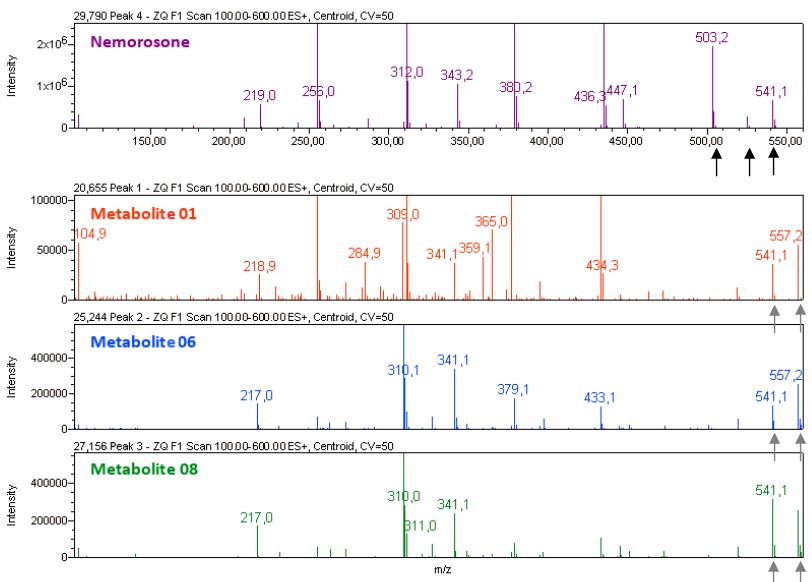

Supplement: Figure S4 — ESI-MS spectra of nemorosone and selected metabolites. ESI-MS spectra were recorded for each peak, and spectra for nemorosone as well as for metabolites M01, M06 and M08 are displayed. The molecule ion peaks of protonated nemorosone (m/z = 503.2) as well as its sodium (m/z = 525.2) and potassium (m/z = 541.1) salts are marked with black arrows. Potentially oxidized nemorosone sodium (m/z = 541.1) and potassium (m/z = 557.2) salts are marked with grey arrows. (TIF) [file pone.0074555.s004.tif]
